# Associations of ALT, AST and ALT/AST ratio with metabolically unhealthy obesity in the elderly

**DOI:** 10.3389/fnut.2025.1513029

**Published:** 2025-03-24

**Authors:** Yuhan Shao, Hailong Zhu, Xin Chen, Enqiang Feng, Chen Chen, Zhugang Shao, Xiaojing Li, Li Liu

**Affiliations:** ^1^Qingdao Municipal Center for Disease Control and Prevention, Qingdao, Shandong, China; ^2^Qingdao Institute of Preventive Medicine, Qingdao, Shandong, China; ^3^Shandong Muhua Medical Technology Co., Ltd., Qingdao, Shandong, China

**Keywords:** metabolically unhealthy obesity, ALT, AST, ALT/AST ratio, elderly

## Abstract

**Aims:**

This study aimed to clarify the effects of alanine aminotransferase (ALT), aspartate aminotransferase (AST) and ALT/AST ratio on metabolically unhealthy obese (MUHO) and to estimate the predictors of MUHO in the elderly.

**Methods:**

19,812 individuals aged 65 years and older from a health check-up in Qingdao, China in 2021 were chosen as subjects in the current study. Binary logistic regression models were performed to evaluate the relationship between ALT, AST, ALT/AST ratio and MUHO. Receiver operating characteristic (ROC) analysis was performed to estimate the predictive value of ALT, AST and ALT/AST ratio for the diagnosis of MUHO.

**Results:**

The risks for MUHO increased across quartiles of ALT level and ALT/AST ratio in both genders. The adjusted odds ratios (ORs) for MUHO in the highest quartile of ALT were 3.20-fold higher than the reference quartile in men and 3.05-fold higher in women. Compared with the first quartile of ALT/AST ratio, the adjusted ORs for MUHO in the highest quartile were 3.64 (95% CI: 3.17–4.19) in men and 3.60 (95% CI: 3.11–4.16) in women, respectively. In ROC curve analysis for predicting MUHO, the area under the ROC curve (AUC) values were 0.63 (*p* < 0.001) for ALT and 0.64 (*p* < 0.001) for ALT/AST ratio in men, and 0.62 (*p* < 0.001) for ALT and 0.64 (*p* < 0.001) for ALT/AST ratio in women. However, AST was not significantly associated with MUHO both in men and in women (*p*>0.05).

**Conclusion:**

ALT and ALT/AST ratio might be considered as two simple and reliable diagnostic indicators for MUHO in the elderly.

## Introduction

Considering the high prevalence of obesity and related metabolic impairments in the population, obesity has become a global health problem (1). Obesity is classified into metabolically healthy obese (MHO) and metabolically unhealthy obese (MUHO) (2–5). MHO individuals present with normal metabolic characteristics, such as normal insulin sensitivity, normal blood pressure, normal serum lipid level and normal liver function, whereas the MUHO individuals are on the contrary (6). Due to differences in race, source of data and the definition for MUHO, the prevalence of MUHO varies considerably in different countries (7). MUHO individuals have higher risks for mortality, cardiovascular events, cancer and worse medical prognosis than those with MHO (8–10). Nonetheless, MHO is a kind of unstable phenotype and is prone to convert to MUHO (11).

For the elderly, traditional MUHO diagnostic criteria might be confounded by age-related physiological changes, such as sarcopenia and insulin sensitivity decline. Furthermore, the presence of comorbidities related to chronic diseases may artificially inflate the prevalence of metabolic abnormalities defined by conventional parameters, ultimately diminishing the specificity of these criteria for accurately identifying MUHO (12). Thus, it is important to identify some other simple biomarkers predicting MUHO to prevent obese individuals to have metabolic derangement. Unlike traditional parameters, alanine aminotransferase (ALT) and aspartate aminotransferase (AST) serve as direct indicators of ectopic fat accumulation and subclinical liver injury, which are integral to the pathophysiology of MUHO but not captured by routine metabolic assessments (13, 14).

In fact, previous studies have reported that ALT and AST levels are linked to MUHO, but the conclusions are controversial (11, 15, 16). One study has demonstrated that the levels of ALT and AST are positively associated with MUHO in both genders (17), while the positive associations are only found in women but not in men in another study (18). Besides, no significant differences in hepatic enzymes are also observed between individuals with MHO and those with MUHO (15). Furthermore, no study has been conducted to analyze the effect of ALT/AST ratio on MUHO in the elderly though the ALT/AST ratio are reported to be significantly related to MUHO in children and adolescents (19). Therefore, the purposes of our study are: (1) to further clarify the effects of ALT and AST on MUHO; (2) to estimate the relationship between ALT/AST ratio and MUHO for the elderly. In addition, we hypothesize that ALT, AST and ALT/AST ratio might be predictors for MUHO.

## Methods

### Subjects and design

The data in the present study were collected face-to-face by trained doctors and nurses in a health check-up for individuals aged over 65 years old in five community centers in Qingdao, China in 2021. The elderly who participated in the free health examinations underwent:(1) Questionnaire survey: answering questions about demographic issues, lifestyle habit (including the frequency of physical activity: never, 1–3 days/week, ≥3 days/week) and dietary behavior (including types of food and ratio of vegetable to meat); (2) Anthropometric measurements: height, weight, waist circumference (WC), systolic blood pressure (SBP) and diastolic blood pressure (DBP); and (3) laboratory measurements: fasting plasma glucose (FPG), ALT, AST, serum total cholesterol (TC), triglycerides (TG) and high-density lipoprotein cholesterol (HDL-C) concentrations.

The participants wore light clothes and removed their shoes for height and weight assessment. Body mass index (BMI) was calculated as weight in kilograms divided by height in meters squared (kg/m^2^). After at least a 5-min rest, three consecutive blood pressure readings from the right arm of each participant were recorded at least 30s apart, and the mean of the three readings was used in our data analysis. WC was measured at the minimal abdominal girth between the costal margin and iliac crest using plastic tape in standing position. All laboratory indicators were measured on a Roche Modular Analytics System (Roche Diagnostics, Basel, Switzerland) and tested in a clinical laboratory using standard clinical protocols.

This study was conducted in accordance with the declaration of Helsinki and approved by the Ethic Committee of Qingdao Municipal Center for Disease Control and Prevention. Written informed consent was obtained prior to subjects’ enrollment in the study.

Exclusion criteria were: (1) BMI<25 kg/m^2^ (18); (2) self-reported dysthyroidism; (3) type 1 diabetes; (4) self-reported congenital cardiovascular diseases; (5) secondary hypertension; (6) self-reported chronic kidney disease; (7) blood pressure, blood glucose or blood lipid altering medications; (8) medications that can alter liver enzymes; (9) heavy alcohol intake (≥40 g alcohol per day); (10) incomplete data on ALT, AST, WC TG, FPG, HDL-C, LDL, SBP and DBP. Altogether, 19,812 subjects were included in the analysis.

### Definitions

In the current study, MHO was defined as having less than three following risk factors, while MUHO was defined as having at least three following risk factors (20, 21):

(1) TG ≥ 1.7 mmol/L or use of lipid-lowering drugs, (2) SBP/DBP ≥130/85 mmHg or use of antihypertensive drugs, (3) FPG ≥5.6 mmol/L or use of medications for diabetes, (4) HDL-C ≤ 1.0/1.3 mmol/L for men/women, and (5) WC ≥90/80 cm for men/women. Balanced diet habit was defined as consuming adequate types of food with an appropriate ratio of vegetable to meat intake (22).

### Statistical analysis

Continuous variables were reported as mean ± standard deviation (SD) and categorical data were presented as number (N) and percentage (%). Quantitative and qualitative variables were compared between the MHO and MUHO groups using independent samples *t*-test and *χ*^2^ test in male and female subjects, respectively. The odds ratios (ORs) with 95% confidence intervals (95% CI) were estimated according to increasing quartiles of ALT, AST and ALT/AST ratio by binary logistic regression models. Receiver operating characteristic (ROC) analysis was conducted for the estimation of ALT, AST and ALT/AST ratio in predicting the diagnosis of MUHO. All statistical analyses were performed using SPSS 20.0 (IBM, Chicago, IL, USA). A two-tailed *p* < 0.05 was considered as statistically significant.

## Results

### General characteristics of the participants

There were 7,846 men and 11,966 women among the total 19,812 participants aged over 65 years old and the average age was (73.59 ± 4.99) years. As shown in Table 1, men had higher level of WC than women (*p* = 0.043), while women had higher HDL-C level than men (*p* = 0.005). There were no significant differences in age, BMI, WC, TG, SBP, DBP, FPG, ALT, AST and ALT/AST ratio between men and women.

**Table 1:** General characteristics of the study population by gender.

Variables	Total *n* = 19,812	Men *n* = 7,846	Women *n* = 11,966	*p* value
Age (y)	73.59 ± 4.99	73.60 ± 5.01	73.59 ± 4.98	0.898
Education				
Illiteracy	4,770 (24.10)	1,969 (25.10)	2,801 (23.40)	<0.001
Primary	13,881 (70.10)	5,531 (67.90)	8,550 (71.50)	
Secondary	975 (4.90)	455 (5.80)	520 (4.30)	
Senior or higher	186 (0.90)	91 (1.20)	95 (0.80)	
Current smoking	1,728 (8.70)	654 (8.30)	1,074 (9.00)	0.118
Current drinking	1,906 (9.60)	744 (9.50)	1,162 (9.70)	0.594
Married	16,839 (85.00)	6,665 (84.90)	10,174 (85.00)	0.883
Physical activity				
Never	13,708 (69.20)	5,329 (67.90)	8,379 (70.00)	0.005
1–3 days/week	1,157 (5.80)	464 (5.90)	693 (5.80)	
≥3 days/week	4,947 (25.0)	2,053 (26.20)	2,894 (24.20)	
Balanced diet habit	1,828 (9.20)	700 (8.90)	1,128 (9.40)	0.230
BMI (kg/m^2^)	28.25 ± 2.63	28.27 ± 2.63	28.24 ± 2.63	0.450
WC (cm)	91.59 ± 7.56	91.72 ± 7.60	91.50 ± 7.52	0.043
TG (mmol/L)	1.74 ± 1.17	1.76 ± 1.12	1.73 ± 1.20	0.153
SBP (mmHg)	152.73 ± 20.14	152.74 ± 20.19	152.73 ± 20.10	0.955
DBP (mmHg)	82.81 ± 11.28	82.81 ± 11.27	82.81 ± 11.28	0.976
FPG (mmol/L)	6.75 ± 2.07	6.75 ± 2.09	6.74 ± 2.06	0.621
HDL-C (mmol/L)	1.46 ± 0.29	1.45 ± 0.29	1.46 ± 0.29	0.005
ALT (U/L)	24.12 ± 13.21	24.06 ± 13.04	24.15 ± 13.31	0.627
AST (U/L)	20.99 ± 8.61	20.88 ± 8.28	21.06 ± 8.81	0.135
ALT/AST ratio	1.16 ± 0.42	1.16 ± 0.42	1.15 ± 0.42	0.436

Data were presented as mean ± SD for continuous variables, and n (%) for categorical variables.

*p values in *t* tests for differences in means or Chi-square tests for differences in proportions.

### Comparison of anthropometric and biochemical measures in MHO and MUHO

As detailed in Table 2, the prevalence of MUHO was 60.38% in men and 81.64% in women, respectively. MUHO group had lower levels of HDL-C than MHO group in both genders. However, the levels of BMI, WC, TG, SBP, DBP, FPG, ALT, AST and ALT/AST ratio were all higher in MUHO group compared to the MHO group both in men and in women (*p*<0.001).

**Table 2:** Anthropometric and biochemical measures by metabolic status.

Variables	Men		Women	
	MHO *n* = 3,109	MUHO *n* = 4,737	MHO *n* = 2,198	MUHO *n* = 9,768
Age (y)	73.68 ± 5.13	73.54 ± 4.94	73.57 ± 5.04	73.60 ± 4.97
BMI (kg/m^2^)	27.64 ± 2.35	28.69 ± 2.72^▲▲^	27.93 ± 2.50	28.31 ± 2.68^▲▲^
WC (cm)	87.85 ± 6.65	94.26 ± 7.11^▲▲^	89.32 ± 7.96	91.99 ± 7.33^▲▲^
TG (mmol/L)	1.31 ± 0.73	2.05 ± 1.23^▲▲^	1.17 ± 0.42	1.86 ± 1.28^▲▲^
SBP (mmHg)	147.9 ± 21.26	155.92 ± 18.79^▲▲^	143.32 ± 22.59	154.84 ± 18.86^▲▲^
DBP (mmHg)	80.84 ± 11.11	84.10 ± 11.19^▲▲^	79.64 ± 11.43	83.53 ± 11.12^▲▲^
FPG (mmol/L)	6.03 ± 1.71	7.23 ± 2.18^▲▲^	5.50 ± 1.08	7.02 ± 2.13^▲▲^
HDL-C (mmol/L)	1.50 ± 0.9	1.42 ± 0.28^▲▲^	1.59 ± 0.26	1.43 ± 0.29^▲▲^
ALT (U/L)	21.24 ± 11.38	25.91 ± 13.71^▲▲^	20.46 ± 10.00	24.98 ± 13.82^▲▲^
AST (U/L)	20.23 ± 7.71	21.25 ± 8.61^▲^	20.27 ± 6.73	21.24 ± 9.21^▲▲^
ALT/AST ratio	1.06 ± 0.39	1.23 ± 0.43^▲▲^	1.01 ± 0.34	1.19 ± 0.42^▲▲^

Data were presented as mean ± SD for continuous variables, and *n* (%) for categorical variables.

^▲p < 0.05, ▲▲p < 0.001: MHO subgroup vs. MUHO subgroup.^

### The correlations between hepatic enzymes and MUHO risk

As shown in Tables 3, 4, the adjusted ORs were 1.59 (95% CI: 1.39–1.81), 2.65 (95% CI: 2.31–3.03) and 3.20 (95% CI: 2.78–3.69) for the second, the third and the fourth quartile of ALT in men in model 3, and the respective ORs were 1.35 (95% CI: 1.19–1.52), 2.00 (95% CI: 1.75–2.28) and 3.05 (95% CI: 2.64–3.52) for Q_2_, Q_3_ and Q_4_ group in women. For men, the fully adjusted ORs for MUHO were 1.67 (95% CI: 1.47–1.90), 2.63 (95% CI: 2.30–3.00) and 3.64 (95% CI: 3.17–4.19) in the second, the third and the fourth quartile of ALT/AST ratio as compared to the first quartile. For women, we also observed a trend of increasing ORs for MUHO with increasing quartiles of ALT/AST ratio in all models. However, increasing AST level was not significantly associated with MUHO in both genders after adjusting for potential confounders (*p*>0.05).

**Table 3:** Logistic regression analysis of association of ALT, AST and ALT/AST ratio with the risk of MUHO in obese men.

ALT (U/L)	*N* (%)	Model 1 OR (95% CI) *p* value	Model 2 OR (95% CI) *p* value	Model 3 OR (95% CI) *p* value
Q_1_ (<16)	1,769 (22.50)	1	1	1
Q_2_ (16–21)	1,976 (25.20)	1.57 (1.38–1.79)<0.001	1.59 (1.40–1.81)<0.001	1.59 (1.39–1.81)<0.001
Q_3_ (21–28.35)	2,140 (27.30)	2.65 (2.33–3.02)<0.001	2.70 (2.36–3.08)<0.001	2.65 (2.31–3.03)<0.001
Q_4_ (≥28.35)	1,961 (25.00)	3.32 (2.90–3.80)<0.001	3.39 (2.95–3.89)<0.001	3.20 (2.78–3.69)<0.001
AST (U/L)				
Q_1_ (<18)	1,588 (20.20)	1	1	1
Q_2_ (18–21)	1,780 (22.70)	0.95 (0.83–1.09) 0.452	0.94 (0.83–1.09) 0.438	0.94 (0.82–1.08) 0.415
Q_3_ (21–25)	2,192 (27.90)	1.06 (0.93–1.21) 0.356	1.06 (0.93–1.21) 0.365	1.05 (0.92–1.20) 0.489
Q_4_ (≥25)	2,286 (29.10)	0.99 (0.88–1.13) 0.944	0.99 (0.88–1.13) 0.922	1.01 (0.88–1.15) 0.937
ALT/AST ratio				
Q_1_ (<0.89)	1,986 (25.30)	1	1	1
Q_2_ (0.89–1.11)	1,941 (24.70)	1.66 (1.47–1.89)<0.001	1.68 (1.48–1.91)<0.001	1.67 (1.47–1.90)<0.001
Q_3_ (1.11–1.37)	1,954 (24.90)	2.63 (2.31–2.99)<0.001	2.68 (2.35–3.05)<0.001	2.63 (2.30–3.00)<0.001
Q_4_ (≥1.37)	1,965 (25.00)	3.62 (3.16–4.14)<0.001	3.70 (3.23–4.24)<0.001	3.64 (3.17–4.19)<0.001

Model 1: unadjusted.

Model 2: adjusted for age.

Model 3: adjusted for age, education, married status, current smoking, current drinking, physical activity, diet habit and BMI.

**Table 4:** Logistic regression analysis of association of ALT, AST and ALT/AST ratio with the risk of MUHO in obese women.

ALT (U/L)	*N* (%)	Model 1 OR (95% CI) *p* value	Model 2 OR (95% CI) *p* value	Model 3 OR (95% CI) *p* value
Q_1_ (<16)	2,621 (21.90)	1	1	1
Q_2_ (16–21)	3,123 (26.10)	1.34 (1.19–1.51)<0.001	1.36 (1.20–1.53)<0.001	1.35 (1.19–1.52)<0.001
Q_3_ (21–28)	2,994 (25.00)	1.99 (1.74–2.26)<0.001	2.03 (1.78–2.32)<0.001	2.00 (1.75–2.28)<0.001
Q_4_ (≥28)	3,228 (27.00)	3.04 (2.64–3.50)<0.001	3.12 (2.71–3.60)<0.001	3.05 (2.64–3.52)<0.001
AST (U/L)				
Q_1_ (<18)	2,922 (24.40)	1	1	1
Q_2_ (18–21)	2,774 (23.20)	0.94 (0.82–1.07) 0.346	0.94 (0.82–1.07) 0.348	0.94 (0.82–1.07) 0.354
Q_3_ (21–25)	3,109 (26.00)	0.99 (0.87–1.14) 0.895	0.99 (0.87–1.13) 0.896	0.99 (0.87–1.13) 0.873
Q_4_ (≥25)	3,161 (26.40)	1.01 (0.88–1.15) 0.913	1.01 (0.88–1.15) 0.915	1.00 (0.88–1.14) 0.975
ALT/AST ratio				
Q_1_ (<0.89)	2,922 (24.40)	1	1	1
Q_2_ (0.89–1.11)	2,774 (23.20)	1.50 (1.33–1.69)<0.001	1.53 (1.35–1.72)<0.001	1.51 (1.34–1.70)<0.001
Q_3_ (1.11–1.35)	3,109 (26.00)	2.25 (1.97–2.56)<0.001	2.30 (2.02–2.62)<0.001	2.27 (1.99–2.59)<0.001
Q_4_ (≥1.35)	3,161 (26.40)	3.55 (3.08–4.10)<0.001	3.66 (3.16–4.23)<0.001	3.60 (3.11–4.16)<0.001

Model 1: unadjusted.

Model 2: adjusted for age.

Model 3: adjusted for age, education, married status, current smoking, current drinking, physical activity, diet habit and BMI.

### ROC analysis

In men, using 18.10 U/L as the cutoff point for ALT yielded a ROC curve with an area under the ROC curve (AUC) of 0.63 (95% CI: 0.62–0.65, *p* < 0.001), a sensitivity of 71.93% and a specificity of 49.85%. If ALT/AST ratio was 1.04 or higher, MUHO was detected with 68.76% sensitivity and 54.66% specificity (AUC = 0.64, *p* < 0.001). In women, the AUC of ALT for predicting MUHO were 0.62 (95% CI: 0.61–0.65, *p* < 0.001) and the optimal cut-off was 18.05 U/L (sensitivity: 66.86%, specificity: 53.78%). The AUC of ALT/AST ratio for MUHO were 0.64 (95% CI: 0.62–0.65, *p* < 0.001) and the optimal cut-off was 1.07 (sensitivity: 58.99%, specificity: 64.89%). However, AST was not useful on the diagnosis of MUHO in both genders (*p*>0.05) (Supplementary Figures 1, 2).

## Discussion

The present study showed that ALT level and ALT/AST ratio were both positively associated with MUHO in the elderly. Furthermore, ALT level and ALT/AST ratio might be considered as two predictive indicators for the diagnosis of MUHO. To the best of our knowledge, this is the first study to examine the effect of ALT/AST ratio on MUHO for the elderly.

Previous studies demonstrated that MHO individuals had significantly lower ALT concentration compared to MUHO subjects (3, 17). High ALT level could increase the risk of MUHO in obese individuals (17, 18, 23). In addition, many studies revealed that ALT was independently linked to metabolic disorders for general population (24, 25). Even in the normal range, individuals with elevated ALT level had an increased risk of cardiovascular risks (20, 26, 27). Our study also showed that ALT concentration was positively related to MUHO in obese men and women as compared to MHO, though AST was not linked to MUHO for both genders. ALT levels in the second quartile and in the third quartile group were also in the normal range (<40 U/L), and subjects in these groups all had higher risks for MUHO than the reference quartile group in our study, which was in line with previous published studies. MHO was an unstable phenotype and was prone to convert to the MUHO state (28). Therefore, obese individuals in the elderly with ALT level higher than the cut-off (18.10/18.05 U/L for men/women) should be paid more attention to prevent the occurrence of metabolic impairment.

Up to now, only one study choosing individuals aged 6–18 years as subjects reported the impact of ALT/AST ratio on MUHO, and this study found that ALT/AST ratio was significantly associated with MUHO in children and adolescents (19). Our study also indicated that ALT/AST ratio was independently and significantly related to MUHO in the elderly. Obese individuals with high ALT/AST ratio (≥1.04/1.07 for men/women) were at an increased risk of transitioning to an unhealthy metabolic status. Thus, ALT/AST ratio might be used as another indicator of poor metabolic control for obese people in the elderly. Nevertheless, the AUCs of ALT and ALT/AST ratio for MUHO were both lower than 0.70 in men and in women though the values were significant. Further investigations on ALT or ALT/AST ratio predicting MUHO are needed for the elderly.

The association between elevated ALT levels or the ALT/AST ratio and the risk of metabolic disorders may be driven by insulin resistance (IR) (25, 29). Elevated ALT in MUHO marked hepatic lipid toxicity from visceral adipose-driven free fatty acid overflow. Lipotoxic metabolites and inflammation disrupted insulin signaling, perpetuating systemic IR. IR could result in steatosis and fibrosis by enhancing fatty acid-oxidation and oxidative stress, and then led to metabolic impairment (30, 31). Moreover, ALT was an indicator of non-alcoholic fatty liver disease (NAFLD) (13, 14). The level of ALT increased extensively in NAFLD, and NAFLD could exacerbate IR by impairing hepatic insulin signaling and trigger the release of pro-inflammatory cytokines and vasoactive mediators, which collectively promoted systemic inflammation and metabolic abnormalities (32, 33). Furthermore, MUHO individuals had 54% more fat accumulation in the liver than those with MHO (34). More liver fat content could also induce IR and further other metabolic disorders (26). However, we found AST was not linked to MUHO, likely due to AST’s lower liver specificity than ALT. AST was widely distributed in other tissues, making its levels susceptible to non-hepatic influences (35). In contrast, ALT was predominantly localized in the liver, making it a more specific marker for hepatic metabolic abnormalities, which were central to MUHO (36).

The study has some limitations. First, the sample was not obtained from a random sample. Second, it could not demonstrate a causal relationship because of its cross- sectional design. Further cohort or molecular biology studies are needed to explore the relationship between ALT, AST, ALT/AST ratio and MUHO. Third, the results may not be directly generalizable to other populations since we focused on the individuals aged 65 years and older.

## Conclusion

ALT and ALT/AST ratio were both independently and significantly related to MUHO in the elderly. ALT and ALT/AST ratio might be used as two simple clinical indicators for identifying MUHO individuals in the elderly. More comprehensive diagnostic criteria for MUHO based on ALT, ALT/AST ratio and other indicators could be formulated. Furthermore, ALT and ALT/AST ratio could be incorporated into routine blood test for the elderly with obesity to label those at high risk of MUHO, even before significant metabolic abnormalities appear.

## Data Availability

The original contributions presented in the study are included in the article/Supplementary material, further inquiries can be directed to the corresponding authors.
