# Supplementary figures and images for: Associations of ALT, AST and ALT/AST ratio with metabolically unhealthy obesity in the elderly
Source: Front Nutr. 2025 Mar 24;12:1513029. doi: 10.3389/fnut.2025.1513029 (PMC11973076; doi:10.3389/fnut.2025.1513029)

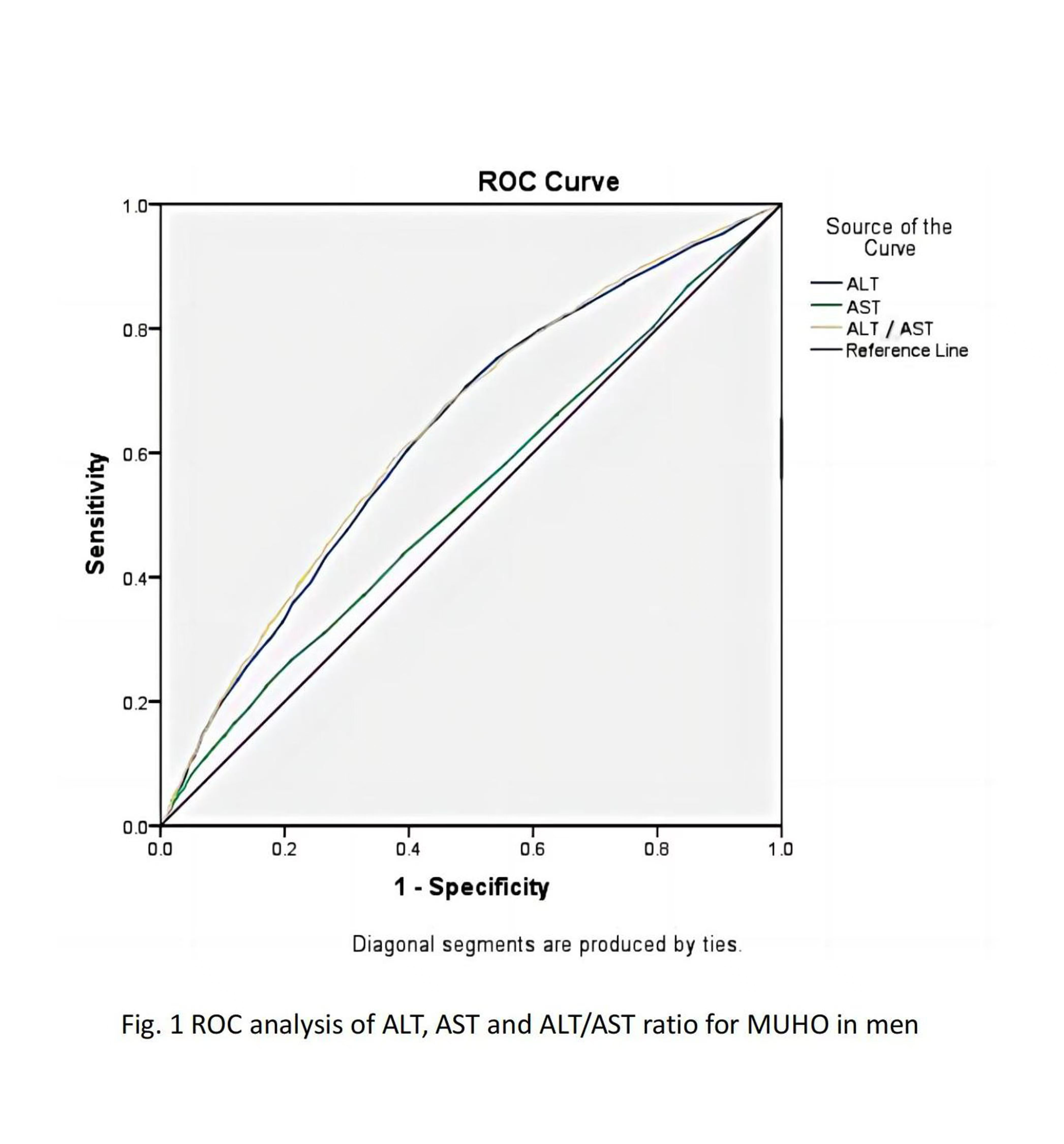

Supplement: Supplementary file 1 [file Image_1.jpeg]
